# Validity, reliability, and diagnostic accuracy of the InGrip digital dynamometer compared with the Jamar hydraulic model in older adults

**DOI:** 10.1007/s41999-025-01403-9

**Published:** 2026-02-26

**Authors:** Luis Polo-Ferrero, Roberto Méndez-Sánchez, Javier Martín-Vallejo, Javier Torres-Alonso, Marta Beatriz Carrera-Villegas, Fausto J. Barbero-Iglesias, Alfonso J. Cruz-Jentoft, Beatriz Montero-Errasquín

**Affiliations:** 1 Departamento de Enfermería y Fisioterapia, Universidad de Salamanca, 37007 Salamanca, Spain; 2 Instituto de Investigación Biomédica de Salamanca (IBSAL), 37007 Salamanca, Spain; 3 Departamento de Estadística, Facultad de Medicina, Universidad de Salamanca, 37007 Salamanca, Spain; 4 Servicio de Geriatría. Hospital Universitario Ramón y Cajal (IRYCIS), 28034 Madrid, Spain

**Keywords:** Handgrip strength, Digital dynamometer, InGrip device, Sarcopenia, Diagnostic accuracy, Older adults

## Abstract

**Introduction:**

Handgrip strength is essential for diagnosing sarcopenia and assessing muscle function in older adults. The Jamar hydraulic dynamometer, backed by strong clinical and epidemiological data, is widely used; however, weight and calibration requirements may limit use. Digital models such as InGrip offer stable calibration and ergonomic design. This study aimed to validate accuracy, reliability, and diagnostic performance of InGrip versus the Jamar.

**Methods:**

A cross-sectional, randomized, single-center study of 415 community-dwelling older adults (mean age: 75.3 ± 6.5 years). Handgrip strength was measured with both devices per the Southampton protocol. Correlation and agreement (Bland–Altman, ICC) between InGrip and the reference dynamometer (Jamar) were analyzed, and diagnostic capacity via ROC curves, estimating sensitivity, specificity, and predictive values for low handgrip strength per EWGSOP2 criteria.

**Results:**

The InGrip dynamometer showed strong correlation with the Jamar dynamometer (r = 0.95) and excellent agreement (ICC = 0.94), intra-observer (ICC = 0.96), and inter-observer (ICC = 0.96), with a mean bias of − 0.37 kg and limits of agreement from – 4.45 to 3.72 kg. Its diagnostic capacity was high (AUC women = 0.968; AUC men = 0.982). Optimal cutoffs were ≤ 18 kg for women (sensitivity = 92.6%, specificity = 90.5%) and ≤ 27.5 kg for men (sensitivity = 96%, specificity = 93%).

**Conclusion:**

The InGrip digital dynamometer showed excellent validity, reliability, and diagnostic accuracy compared to the Jamar in community-dwelling older adults, with sex-specific cutoffs aligned with EWGSOP2 criteria, supporting its use as a practical alternative for strength assessment in research and clinical practice.

**Trial registration:**

ClinicalTrials.gov NCT06623019.

**Supplementary Information:**

The online version contains supplementary material available at 10.1007/s41999-025-01403-9.

## Key summary points

**Aim:**

To evaluate the validity, reliability, and diagnostic accuracy of the InGrip digital dynamometer compared with the Jamar hydraulic model in community-dwelling older adults.

**Findings:**

The InGrip device showed excellent correlation and agreement with the Jamar dynamometer, with high intra- and inter-rater reliability. It demonstrated outstanding diagnostic performance with sex-specific cutoff values closely aligned with EWGSOP2 criteria.

**Message:**

The InGrip digital dynamometer is a valid, reliable, and clinically useful alternative to the Jamar for assessing handgrip strength in older adults.

**Supplementary Information:**

The online version contains supplementary material available at 10.1007/s41999-025-01403-9.

## Introduction

Handgrip strength (HGS) is a well-established functional marker and the primary diagnostic criterion for sarcopenia across international consensus definitions [1–6]. Low HGS independently predicts disability, falls, hospitalization, institutionalization, and mortality, regardless of clinical or sociodemographic factors [7–9]. It is widely regarded as a surrogate of overall muscle strength and a key measure for monitoring functional decline and frailty [10].

The Jamar hydraulic dynamometer has historically served as the reference instrument for HGS assessment and is the model from which the most robust clinical and epidemiological evidence has been derived [11]. However, its design poses notable limitations in older populations: a rigid grip with broad adjustment intervals, a heavy metallic structure (1.4 kg), the requirement for periodic calibration with associated costs and downtime, and potential observer error from analog readings [12]. Although relatively affordable, these drawbacks reduce feasibility and reliability in both clinical and research settings [7, 11]. Consequently, digital alternatives have been developed, many of which demonstrate strong correlations with the Jamar device [13–20].

However, as validation studies have shown heterogeneous results across digital dynamometers—some proving highly reliable and others not—it remains essential to validate each new model before clinical use. The InGrip dynamometer incorporates electronic sensors and a non-hydraulic system, thereby eliminating the risk of decalibration. It offers precise digital outputs (0.1 kg increments), lighter weight (650 g), and an ergonomic, adaptable grip suitable for individuals with pain or functional limitations. In addition, its software allows integration with other functional and anthropometric assessments, facilitating standardized sarcopenia evaluation [21].

This study aimed to validate the InGrip dynamometer by examining its accuracy, reliability, and predictive validity for HGS measurement, as well as its ability to detect low values based on the European Working Group on Sarcopenia in Older People (EWGSOP2) thresholds, in comparison with the Jamar model, in a large sample of community-dwelling older adults. Rigorous validation of this device is essential prior to its adoption in clinical practice.

## Methods

### Study design

This cross-sectional, randomized, single-center study followed the Standards for Reporting Diagnostic Accuracy Studies (STARD) guidelines [22]. Ethical approval was obtained from an accredited ethics committee (PI code: ICPS-I 24 2050). All participants received study information and provided written informed consent. Procedures complied with the Declaration of Helsinki [23]. This study was prospectively registered at ClinicalTrials.gov (Identifier: NCT06623019) prior to the initiation of participant recruitment.

The study aimed to evaluate HGS in older adults using the Jamar dynamometer and the InGrip device. The primary objective was to compare HGS values obtained with the Jamar model, assess the accuracy, reliability, and predictive validity of the InGrip device, and determine its ability to detect low HGS based on EWGSOP2 cutoffs. To minimize potential order effects, participants were assessed using both devices following a randomized cross-over sequence of device order. In addition, to assess intra- and inter-rater reliability, a subsample was selected by systematically including the first four men and four women evaluated each day, ensuring balanced and consecutive inclusion independent of clinical characteristics.

### Participants

Participants were community-dwelling adults aged 60 years and older enrolled in the Programa de Revitalización Geriátrica (PReGe) at the Facultad de Enfermería y Fisioterapia, Universidad de Salamanca, where older adults are evaluated annually. All participants were informed about the study and provided written informed consent prior to inclusion. Exclusion criteria included inability to understand the instructions, hand pain during the evaluation or in the previous week, and any medical condition contraindicating maximal grip effort. After confirming eligibility, each participant was assigned a unique identifier for coding purposes, and random allocation was used to determine the order in which the two devices were administered. The sequence was randomly generated by an external investigator using a random number table with the Research Randomizer application (https://www.randomizer.org/).

### Procedures and outcomes

HGS was assessed with both devices following the Southampton protocol. Participants were seated in a 43 cm-high chair with backrest and fixed armrests. Forearms rested on the armrests, with the wrist in a neutral position and dorsiflexed 0°–30°, thumb pointing upward, and fingers wrapped around the handle. For the Jamar dynamometer, the second handle position—recommended as the most reliable configuration—was used for all participants unless hand size required the first position. The exact grip span corresponding to the selected Jamar setting was measured in millimeters between the inner surfaces of the handles. The InGrip device features a sliding, lockable handle adjustment system; therefore, its grip was calibrated to match the same span measured on the Jamar for each participant. This ensured that both instruments provided an equivalent hand opening, allowing direct comparability of maximal force outputs. The InGrip handle was additionally aligned to maintain the same wrist-neutral position described in the Southampton protocol [11]. The InGrip is factory-calibrated and uses an internal electronic load-sensing system, therefore not requiring routine external calibration. Manufacturer testing including up to 50,000 repeated measurements showed deviations within ± 1%, supporting stable and consistent handgrip strength assessment. Participants were instructed to squeeze maximally and received verbal encouragement. Three consecutive trials were performed with the dominant hand for each device in randomized order. The highest value was recorded. A 30-s rest was given between repetitions and 3–5 min between devices to minimize fatigue. To assess potential learning or fatigue effects, we subsequently examined whether handgrip performance differed according to the device used first.

Additionally, sociodemographic variables such as age and sex, medical information including the number of medications, and anthropometric measures such as weight, height, body mass index (BMI), and appendicular skeletal muscle mass index (ASMI) were collected using bioelectrical impedance analysis with the TANITA BC-418 device. Functional variables, including the Short Physical Performance Battery (SPPB) and the Five Times Sit-to-Stand Test (5STS), were also recorded.

For inter-rater reliability, a second evaluator (J.T-A) repeated the assessment 30 min after the first. Intra-rater reliability was assessed by repeating the procedure 5–10 days later with the same evaluator (L.P-F). Assessors performing the index test were blinded to the results of the reference standard, and vice versa. Clinical information was not disclosed to the assessors during testing to avoid bias.

### Sample size

Two calculations were performed: one for the overall study and another for the intra- and interobserver validation. Both were based on previous studies validating HGS devices against the Jamar dynamometer [16, 18]. These studies reported excellent intraclass correlation coefficients (ICC > 0.90; ICC 0.95 and 0.93, respectively, with 95% confidence intervals (CIs). Assuming an ICC of 0.90 ± 0.05 and a 5% significance level, 350 participants were required for the main study. For the validation study, using 95% CI widths of 0.22–0.23 from prior work and a mean diagnostic error probability of 0.23, the required sample was 70 participants to assess cutoff point concordance in repeated measures. All calculations were performed with GRANMO v.8.

### Statistical analysis

Descriptive statistics were calculated for all baseline variables, presented for the total sample and stratified by sex. Continuous variables are reported as means ± standard deviation (SD) and categorical variables as frequencies and percentages. The distribution of HGS values from both devices was assessed using the Kolmogorov–Smirnov test and boxplot inspection. To verify that device administration order did not bias results, baseline characteristics and HGS values were compared between the two randomization groups using independent-samples t-tests (or Mann–Whitney U tests when normality assumptions were not met).

Agreement between the Jamar and InGrip dynamometers was evaluated using complementary methods. Pearson’s correlation coefficient assessed linear association, and simple linear regression quantified explained variance (R^2^). Measurement agreement was further examined with Bland–Altman plots, calculating mean bias and 95% limits of agreement with confidence intervals, and line-of-identity plots for visual inspection.

ICC was computed with a two-way mixed-effects model (absolute agreement) to assess inter-device, intra-rater, and inter-rater reliability. ICC values were interpreted as poor (< 0.50), moderate (0.50–0.75), good (0.75–0.90), or excellent (> 0.90) [24]. Paired-sample t-tests were used to evaluate differences between repeated measures when appropriate.

Diagnostic validity of the InGrip dynamometer was analyzed using receiver operating characteristic (ROC) curves. The area under the curve (AUC) was calculated separately for women and men, with EWGSOP2 cutoffs (< 16 kg for women, < 27 kg for men, Jamar model) as the reference standard. Sensitivity, specificity, positive predictive value (PPV), and negative predictive value (NPV) were reported for optimal InGrip cutoff points. 95% confidence intervals for sensitivity and specificity have been calculated using Wolf’s method. All analyses were conducted with IBM SPSS Statistics version 28.0, with significance set at P < 0.05. No analyses of variability in diagnostic accuracy were pre-specified or conducted.

## Results

### Baseline characteristics of the sample

**Fig. 1:**
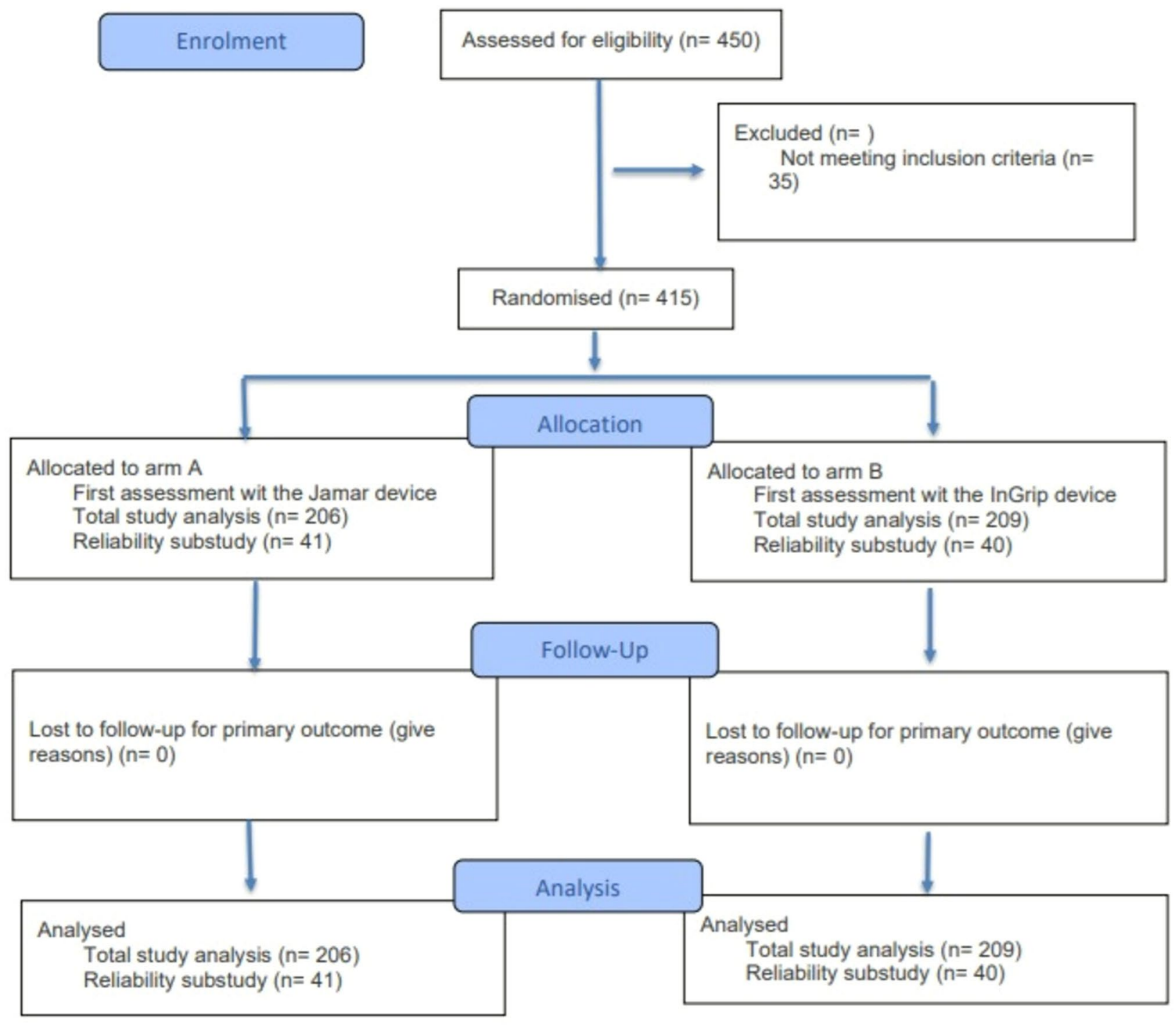
Flow diagram of participants through the study

A total of 450 community-dwelling older adults were screened for eligibility, of whom 35 did not meet the inclusion criteria. Of these, 19 participants were excluded due to hand pain in the previous week, 9 because of recent upper-limb surgery, and 7 due to difficulty following the test instructions. The remaining 415 participants were randomized: 206 allocated to Arm A (Jamar first, including 41 in the reliability substudy) and 209 to Arm B (InGrip first, including 40 in the reliability substudy). No participants were lost to follow-up, all 415 were included in the final analysis (Fig. 1), and no adverse events occurred during the administration of either the index test or the reference standard.

The final sample had a mean age of 75.3 ± 6.5 years, and 83.6% were women (n = 347). Nearly all participants (99.5%, n = 413) reported right-hand dominance. Baseline characteristics are summarized in Table 1. The reliability subsample included 81 participants (mean age 76.1 ± 6.1 years; 49.3% women), selected through a systematic daily inclusion procedure independent of clinical characteristics.

**Table 1:** Baseline characteristics

Variable	Women (n = 347)	Men (n = 68)
Age (years)	75.0 ± 6.4	77.5 ± 6.8
Height (cm)	153 ± 6.0	163 ± 6.0
Weight (kg)	65.0 ± 11.5	74.0 ± 10.0
ASMI (kg/m^2^)	6.0 ± 0.9	6.9 ± 1.0
BMI (kg/m^2^)	27.9 ± 4.6	27.8 ± 3.4
SPPB (points)	11.4 ± 1.1	11.3 ± 1.7
5STS (s)	11.3 ± 2.9	11.4 ± 3.5
HGS_Jamar (kg)	19.5 ± 4.6	32.1 ± 8.2
HGS InGrip (kg)	20.0 ± 4.3	31.7 ± 7.6

Values are expressed as mean ± standard deviation

*5STS* Five Times Sit-to-Stand Test, *ASMI* appendicular skeletal muscle mass index, *BMI* body mass index; cm centimeters, *HGS* handgrip strength, *kg* kilograms, *s* seconds, *SPPB* Short Physical Performance Battery

HGS measured with the Jamar dynamometer was 21.3 ± 6.9 kg for the total sample, 19.5 ± 4.6 kg for women, and 32.1 ± 8.2 kg for men. HGS assessed with the InGrip dynamometer was 21.7 ± 6.4 kg for the total sample, 20.0 ± 4.3 kg for women, and 31.7 ± 7.6 kg for men. Both distributions were normal (p > 0.05), supporting the use of parametric statistical analyses.

No significant differences were observed for any baseline variable, including HGS (p > 0.05), indicating that the order of device administration did not introduce fatigue or sequence effects and confirming the adequacy of the randomization process.

### Correlation and agreement between devices for the measurement of maximal HGS

**Fig. 2:**
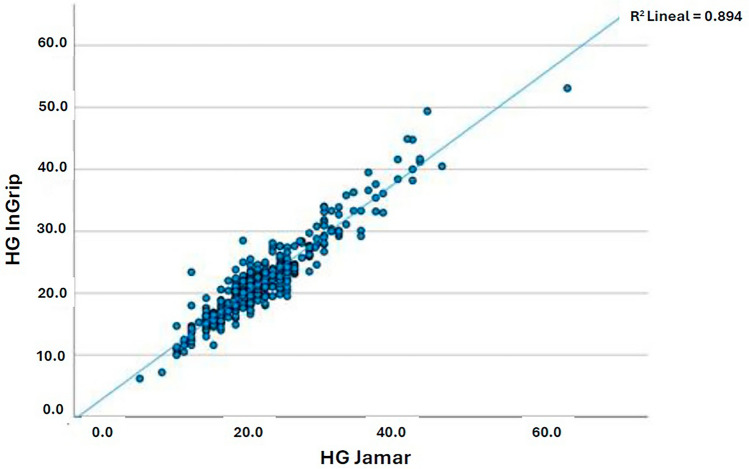
Scatter plot of HGS measurements obtained with the InGrip dynamometer in relation to the Jamar dynamometer

Descriptive statistics showed a mean HGS of 21.3 ± 6.9 kg when measured with the Jamar dynamometer and 21.7 ± 6.4 kg with the InGrip dynamometer. A strong and statistically significant positive correlation was observed between the two devices (r = 0.95; p < 0.001). Linear regression analysis yielded a coefficient of determination (R^2^ = 0.894), indicating that 89.4% of the variance in InGrip dynamometer measurements was explained by the corresponding Jamar model values (Fig. 2). These results demonstrate a high level of agreement between the dynamometers in assessing maximal HGS.

### Agreement between devices: Bland–Altman and line-of-identity analyses

**Fig. 3:**
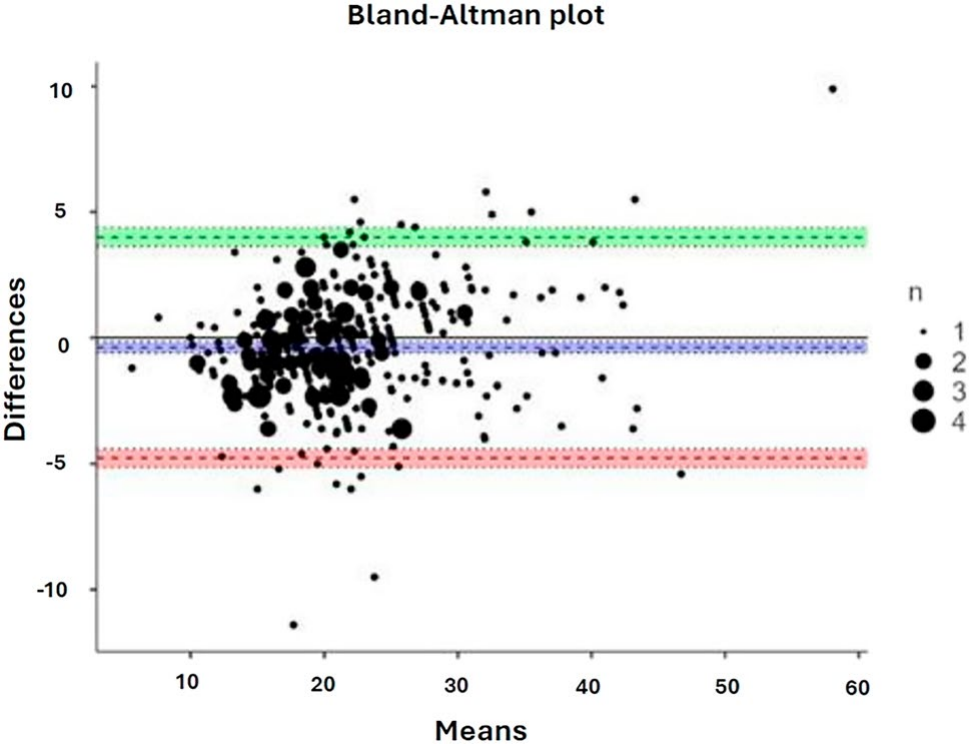
Bland–Altman plot displaying the agreement between HGS measurements. The central line represents the mean difference (bias), while the upper and lower dotted lines represent the 95% limits of agreement

To evaluate agreement between the two devices, we used both a Bland–Altman plot (Fig. 3) and a line-of-identity plot (Fig. S1). The Bland–Altman analysis showed a mean difference (bias) of –0.37 kg (95% CI – 0.57 to – 0.16 kg), indicating that the InGrip dynamometer slightly underestimated HGS compared with the Jamar dynamometer (Table 2). This bias was minimal and did not indicate a meaningful difference between devices. The 95% limits of agreement ranged from – 4.45 kg (95% CI – 4.79 to – 4.11 kg) to 3.72 kg (95% CI 3.37–4.06 kg), within which most values were contained. Consistently, the line-of-identity plot (Fig. S1) demonstrated a close alignment along the 45° line, confirming strong concordance between the two dynamometers.

**Table 2:** Diagnostic classification of the InGrip dynamometer in women (cutoff ≤ 18 kg) and men (cutoff ≤ 27.5 kg)

InGrip classification	EWGSOP2		Total
	Low HGS	Normal HGS	
Women			
Low HGS	87	24	111
Normal HGS	7	229	236
Total	94	253	347
Men			
Low HGS	24	3	27
Normal HGS	1	40	41
Total	25	43	68

Values are expressed as the number of participants (*n*)

*EWGSOP2* European Working Group on Sarcopenia in Older People 2, *HGS* handgrip strength

### Agreement and reliability: intraclass correlation coefficient (ICC)

The ICC analysis demonstrated excellent agreement between HGS measurements obtained with the Jamar and InGrip dynamometers (ICC = 0.94; 95% CI 0.93–0.96), indicating high reproducibility between devices. For intra-rater reliability, the ICC for the highest HGS measurement was 0.96 (95% CI 0.95–0.97), while inter-rater reliability was also 0.96 (95% CI 0.95–0.96). Paired-sample t-tests revealed no statistically significant differences between measurements (inter-device p = 0.203; intra-rater p = 0.504; inter-rater p = 0.272), indicating the absence of systematic bias. Overall, these results confirm the high precision and reproducibility of the InGrip dynamometer compared with the Jamar model.

### Diagnostic cutoff values and predictive validity of the InGrip dynamometer for identifying low muscle strength

ROC curve analysis demonstrated excellent diagnostic accuracy of the InGrip dynamometer for detecting low muscle strength in both sexes. The AUC was 0.968 (95% CI 0.952–0.993) for women and 0.982 (95% CI 0.962–1.000) for men, indicating high precision in distinguishing individuals with and without low muscle strength.

**Fig. 4:**
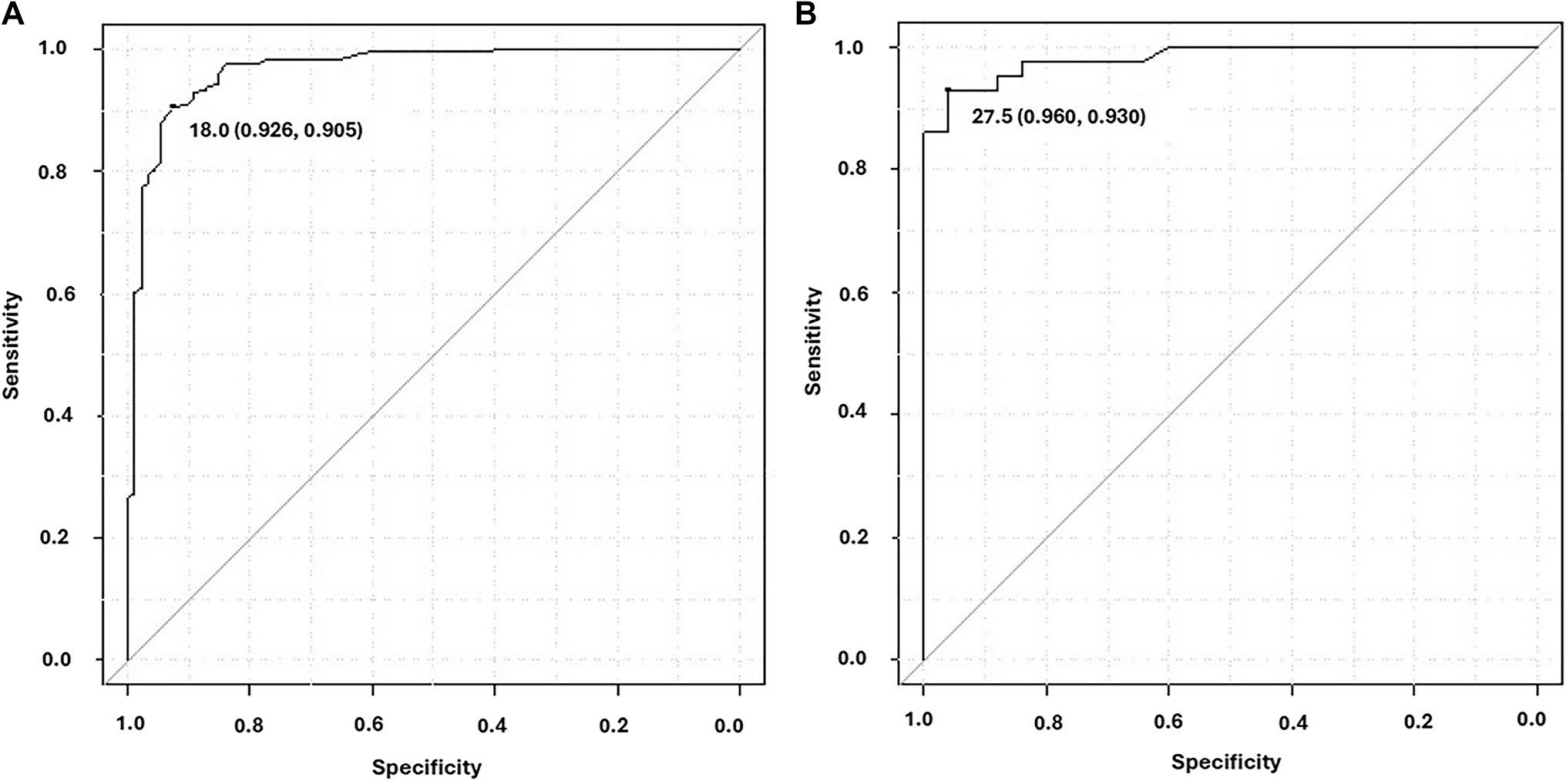
ROC curves for detecting low muscle strength according to EWGSOP2 criteria using the InGrip dynamometer. (**A**) ROC curve for women showing an optimal cutoff point of ≤ 18 kg. (**B**) ROC curve for men showing an optimal cutoff point of ≤ 27.5 kg

In women, the optimal cutoff point was ≤ 18 kg, with a sensitivity of 92.6% (95% CI 85.3–97.0%) and a specificity of 90.5% (95% CI 86.2–93.8%) (Fig. 4A). In men, the optimal cutoff point was ≤ 27.5 kg, with a sensitivity of 96.0% (95% CI 80.0–100.0%) and a specificity of 93.0% (95% CI 81.0–99.0%) (Fig. 4B).

Predictive values further supported the diagnostic validity of the InGrip dynamometer. In women, the PPV was 78.4% (87/111), indicating that 78.4% of those classified as having low handgrip strength by the device were confirmed by the reference standard, while the NPV was 97.0% (229/236), reflecting a high probability of correctly identifying non-frail individuals. In men, the PPV was 88.9% (24/27) and the NPV was 97.6% (40/41), confirming excellent accuracy in both detecting low handgrip strength and excluding it when values were above the cutoff. A misclassification analysis was conducted using EWGSOP2 thresholds as the reference standard. In women, the InGrip cutoff of ≤ 18 kg yielded 87 true positives, 24 false positives, 229 true negatives, and 7 false negatives (false-negative rate: 7.4%; false-positive rate: 9.5%). In men, the sex-specific cutoff of ≤ 27.5 kg produced 24 true positives, 3 false positives, 40 true negatives, and 1 false negative (false-negative rate: 4.0%; false-positive rate: 7.0%). Overall, misclassification rates were low in both sexes—particularly for false negatives—supporting the diagnostic safety of applying these sex-specific cutoff values in clinical screening.

## Discussion

This study provides strong evidence supporting the validity and clinical applicability of the InGrip dynamometer as an alternative to the Jamar device for assessing HGS in community-dwelling older adults. The close correlation and excellent agreement observed between the two instruments confirm the consistency of the InGrip relative to the reference standard. Furthermore, both intra- and inter-rater reliability were excellent, underscoring the robustness of the device across repeated assessments and different observers.

Sarcopenia, which affects approximately 10–27% of older adults, is a major cause of functional decline, loss of independence, and increased morbidity and mortality [1, 25]. Both the revised EWGSOP2 and the Global Leadership Initiative on Sarcopenia (GLIS) emphasize muscle strength as the primary functional marker, underscoring its diagnostic and prognostic importance. Current consensus criteria provide internationally accepted thresholds for identifying low HGS, which served as a framework for interpreting our findings [1, 26]. Within this context, the InGrip dynamometer demonstrated remarkable diagnostic performance. ROC curve analysis confirmed its excellent ability to discriminate low HGS, and the device showed close alignment with EWGSOP2 recommendations regarding sex-specific cutoff points. High sensitivity and specificity reinforced its clinical accuracy, while Bland–Altman analysis indicated only a minimal bias, reflecting a slight, clinically irrelevant underestimation by the InGrip model. Collectively, these results establish the InGrip dynamometer as a valid and precise alternative to the Jamar, combining strong measurement performance with enhanced usability.

Previous studies have shown that several digital dynamometers demonstrate high concordance with the hydraulic Jamar model, supporting their validity as alternative tools [13–20]. However, models with less comparable grip designs have often demonstrated reduced precision and reliability, failing to achieve excellent agreement [27–29]. In the present study, the limits of agreement indicated a methodologically and clinically acceptable range of variation. Importantly, these values should be interpreted in the context of the inherent variability of maximal voluntary grip effort and the ranges reported in previous validation studies, rather than in absolute terms.

When interpreting the limits of agreement, it is essential to consider their clinical relevance and how they compare with previously published validation studies. Our limits (– 4.45 to 3.72 kg) were comparable to, or narrower than, those reported when digital dynamometers were evaluated against the Jamar standard—such as the ranges of – 4.6 to 3.5 kg and – 7.7 to 9.7 kg reported by Guerra et al. [16], the – 4.1 to 5.6 kg described by Jiménez-Sánchez et al. [20], and the broader variability visually observed in Gripwise validation data [18]. Although this degree of inter-device variability may appear substantial in absolute terms, it aligns with the expected fluctuations of maximal voluntary contraction and with the typical differences observed across dynamometer technologies. Importantly, the variability observed in our study did not materially affect diagnostic classification, thereby supporting the clinical acceptability of the InGrip dynamometer.

The minimal mean bias further reinforces the equivalence of the InGrip model to the Jamar reference standard. Consistent with findings from other validated digital devices, the InGrip dynamometer demonstrated high sensitivity and specificity for identifying low muscle strength according to EWGSOP2 criteria [18], indicating that the inter-device variability captured in the Bland–Altman analysis does not translate into clinically meaningful discrepancies. The strong correlation observed in this large sample of community-dwelling older adults also supports its suitability for early detection of reduced handgrip strength, in line with current recommendations for sarcopenia screening and risk stratification [1].

The sex-specific cutoff values identified in this study (≤ 18 kg for women and ≤ 27.5 kg for men) may reflect the device’s ergonomic grip and slight elastic response, which could facilitate maximal force generation during testing. From a practical perspective, the InGrip also offers several advantages over the Jamar: it is lighter, does not require hydraulic calibration, and incorporates a digital interface that improves usability, reduces reader error, and enhances feasibility in geriatric screening. Manufacturer repeated-load testing of up to 50,000 measurements demonstrated deviations within ± 1%, supporting the long-term measurement stability of the device. Its excellent diagnostic accuracy, together with strong negative predictive values, supports its utility for the early detection of low muscle strength, a key component of sarcopenia. Misclassification rates were low—particularly false negatives—reinforcing its safety for screening contexts where missed cases must be minimized. The modest proportion of false positives, especially in women, remains acceptable in population screening and favors early referral rather than missed diagnoses. Combined with the device’s portability and integration with software for automated reporting, the InGrip appears well suited for use in geriatric units, primary care, and community-based assessment programs.

Handgrip strength was quantified using the highest value of three trials, consistent with the Southampton Protocol, which recommends repeated attempts and recording the maximum effort as the most accurate representation of true maximal voluntary contraction [11]. This approach minimizes the influence of submaximal or familiarization attempts and aligns with the methodology used in most dynamometer validation studies. Although some studies use mean values, the maximal score is widely accepted in clinical and epidemiological research and maintains full comparability with established diagnostic frameworks for sarcopenia [30, 31].

Despite these strengths, several limitations must be acknowledged. First, this study was conducted in a single center with community-dwelling older adults, which may limit generalizability to hospitalized or institutionalized populations. Second, only the dominant hand was assessed, precluding exploration of potential lateral differences. Third, the predominance of female participants may restrict the extrapolation of sex-specific diagnostic thresholds, as the smaller male subgroup reduces the precision of estimates for men. This marked sex imbalance reflects the demographic distribution of community-dwelling older adults, in whom women are typically overrepresented; however, it limits the external validity of the sex-specific findings, particularly for men. The reduced male representation may lead to wider confidence intervals, less stable threshold estimation, and restricted generalizability to broader or more clinically diverse male populations. Future validation studies with more balanced samples are therefore needed to confirm the applicability of the device-specific cutoffs in men and to strengthen the generalizability of our diagnostic estimates.

Nonetheless, the optimal male cutoff identified (≤ 27.5 kg) was practically identical to the EWGSOP2 reference value (27 kg) and demonstrated excellent diagnostic performance (AUC = 0.982), suggesting that the male-specific findings—while less precise—remain clinically meaningful. Importantly, diagnostic accuracy for men remained excellent, supporting the robustness of the proposed threshold while still underscoring the need for confirmation in larger and more sex-balanced cohorts. Although the InGrip dynamometer does not require calibration, further research is needed to confirm its long-term stability compared with the Jamar. Future studies should also examine its performance in condition-specific populations where pain or disability may influence grip measurements, as well as in hospitalized or institutionalized older adults, where functional limitations and clinical complexity may affect accuracy. Longitudinal studies are required to determine whether InGrip measurements can predict adverse outcomes such as falls, disability, or mortality, as has been demonstrated with the Jamar [8].

In addition, because the exclusion criteria required the absence of hand pain, recent surgery, or conditions contraindicating maximal effort, our sample consisted of functionally stable older adults capable of performing a true maximal voluntary contraction. This approach is essential to ensure accurate reliability and agreement testing; however, it may limit generalizability to individuals with acute or chronic conditions that produce pain, inflammation, or impaired motor control. Since these factors primarily affect the user rather than the device, the psychometric properties observed here are expected to remain applicable, although absolute HGS values may differ in clinical populations. Future studies should therefore validate the InGrip device in groups with specific conditions (e.g., osteoarthritis, neurological disease, postoperative states) to fully establish its performance across a broader spectrum of clinical conditions. Beyond the present validation in functionally stable older adults, the potential applicability of the InGrip dynamometer to clinical subgroups warrants further exploration. Given its strong diagnostic accuracy and the integration of its outputs with body composition data through InBody’s analytic software, the device may be particularly useful in populations with cognitive impairment (e.g., dementia) or neurological and functional limitations (e.g., Parkinson’s disease), where standardized and digitally assisted assessments can enhance clinical decision-making. Although our design focused on healthy community-dwelling adults to ensure methodological rigor, future validation in these specific clinical groups is essential to confirm whether the InGrip maintains its diagnostic performance under more complex physiological and functional conditions.

To our knowledge, this is the first study to validate the InGrip dynamometer in a large cohort of community-dwelling older adults, using a randomized device sequence to minimize potential bias. We employed a rigorous methodological approach, addressing all key aspects of device validation, thereby strengthening the reliability of our findings. Overall, our results strongly support the InGrip as a valid, reliable, and practical alternative to the Jamar dynamometer, with high reproducibility, ease of use, and digital integration that make it well suited for routine clinical practice and longitudinal monitoring in older adults. In addition to its diagnostic accuracy and predictive validity, the device’s integrated software enables seamless combination with other functional and anthropometric tests, facilitating automated, comprehensive, and standardized assessments of sarcopenia. We consider the InGrip model a promising instrument aligned with current clinical and public health priorities in aging populations.

## Conclusion

The InGrip dynamometer showed excellent agreement with the Jamar model, with strong correlations, high reliability, and minimal bias in community-dwelling older adults. It demonstrated high diagnostic accuracy with cutoff points consistent with EWGSOP2 thresholds and offers practical advantages such as reduced weight, digital readout, and integrated software. These findings support InGrip as a reliable and clinically relevant alternative for assessing handgrip strength and screening for low muscle strength in the context of sarcopenia, particularly in geriatric and primary care settings. Further studies in other clinical populations may help confirm its applicability across a broader range of health conditions.

## Supplementary Information

Below is the link to the electronic supplementary material.

Supplementary file1 Fig. S1. Line-of-identity plot comparing HGS values obtained with the InGrip and Jamar dynamometers. Each point represents paired measurements for an individual participant. The 45° line indicates perfect agreement. (TIFF 41 KB)

## Data Availability

The datasets used and/or analyzed during the current study are available from the corresponding author upon reasonable request. The full study protocol is available from the corresponding author upon reasonable request.
