# Supplementary figures and images for: Validity, reliability, and diagnostic accuracy of the InGrip digital dynamometer compared with the Jamar hydraulic model in older adults
Source: Eur Geriatr Med. 2026 Feb 26;17(3):1169–79. doi: 10.1007/s41999-025-01403-9 (PMC13309481; doi:10.1007/s41999-025-01403-9)

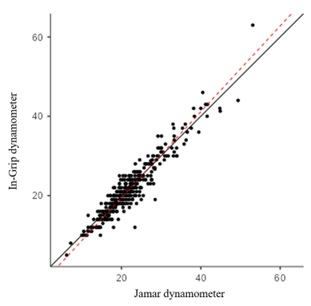

Supplement: Supplementary file 1 — Supplementary file1 Fig. S1. Line-of-identity plot comparing HGS values obtained with the InGrip and Jamar dynamometers. Each point represents paired measurements for an individual participant. The 45° line indicates perfect agreement. (TIFF 41 KB) [file 41999_2025_1403_MOESM1_ESM.tiff]
